# Intranasal Vaccination with Mannosylated Chitosan Formulated DNA Vaccine Enables Robust IgA and Cellular Response Induction in the Lungs of Mice and Improves Protection against Pulmonary Mycobacterial Challenge

**DOI:** 10.3389/fcimb.2017.00445

**Published:** 2017-10-16

**Authors:** Manli Wu, Haoxin Zhao, Min Li, Yan Yue, Sidong Xiong, Wei Xu

**Affiliations:** Jiangsu Provincial Key Laboratory of Infection and Immunity, Institutes of Biology and Medical Sciences, Soochow University, Suzhou, China

**Keywords:** *Mycobacterium tuberculosis*, mannosylated chitosan, DNA, SIgA, poly-functional T cells

## Abstract

Induction of specific humoral and cellular immunity in the lung airways is proposed to be critical for vaccine protection against *Mycobacterium tuberculosis* (*M. tb*). To facilitate airway delivery and antigen targeting to the antigen presenting cells in the alveoli, we employed mannosylated chitosan (MCS) to formulate a multi-T-epitope DNA vaccine, pPES, as an intranasal TB vaccine. MCS-DNA nanoparticles appeared spherical with the average particle sizes as 400 nm. HSP65-specific bronchoalveolar lavage fluid SIgA level was significantly elevated by 4 doses of MCS-pPES intranasal immunization as compared to chitosan (CS)-DNA and BCG vaccine. I.n. immunization with MCS-DNA induced a modest peptide-specific Th1(IFN-γ, TNF-α, and IL-2) response in the spleen, while a potent poly-functional CD4+ T response that largely produced TNF-α and IFN-γ, as well as IL-2 in the lung, qualitatively better than that induced by CS-DNA and BCG vaccination. Such response by i.n. immunization with MCS-DNA provided improved protection in the lung against airway *Mycobacterial bovis* BCG challenge over i.n. CS-DNA and DNA, that is comparable to protection achieved by s.c. BCG vaccination. This enhanced protection was correlated with much greater accessibility of DNA particles to the alveolar macrophages in the lung mediated by man-chitosan. Thus, man-chitosan TB vaccine represents a promising vaccine platform capable of eliciting robust multi-functional T response in the lung mucus and achieving enhanced mucosal immune protection against pulmonary TB.

## Introduction

Tuberculosis (TB) is a potentially fatal contagious disease caused by infection of *Mycobacterium tuberculosis (M.tb)*. Interferon-γ (IFN-γ)+CD3+ T cells have long been thought as dominant immunological cells to clear *M.tb*. Meanwhile, early and locally activated macrophages and neutrophils have important roles in the early control of *M.tb* infection after exposure, although definitive protective mechanism has yet to be obtained (Bhatt et al., 2015; Orr et al., 2015). Since boosting with MVA85A or adenovirus-vectored vaccine intranasally after BCG priming significantly improves protection compared with BCG alone (Santosuosso et al., 2006), a likely limitation of parenteral BCG immunization is considered to be an insufficient induction of effector cells in the lung which is the initial entry site for *M.tb*. Increasing evidence suggests that local immune defense mechanisms in the lung interstitial tissue and bronchoalveolar lavage (BAL) fluid are crucial for protective immunity against *M.tb* (Beverley et al., 2014).

At mucosal epithelium, secretory IgA (SIgA) is the predominant Ig isotype (Brandtzaeg, 2013) and exert superior neutralization activity than IgG for its extracellular immune exclusion effect and pIgR-mediated cytosolic Fc receptor-participated intracellular pathogen neutralization activity (Foss et al., 2015). It has also been demonstrated that lung-resident T and T memory cells with specialized phenotypic and functional properties have an important role in protection against respiratory infections, which depends on dendritic cells (DC) and macrophages-mediated antigen encapsulation and presentation (Beverley et al., 2014). Therefore, immunization via mucosal routes to evoke SIgA and a poly-functional *M.tb* specific T cell immune response in the lung is of great significance for the establishment of protective pulmonary immunity against TB. In this regard, our group has developed a cationic polysaccharides chitosan delivered DNA construct carrying multi-T epitopes grafted into HSP65 scaffold (pPES) which leads to enhanced induction of pulmonary immunity and anti-TB protection (Ai et al., 2013; Wu et al., 2016).

Chitosan and chitosan derivatives have been developed for DNA delivery systems because of their cationic charge, biodegradability and biocompatibility, as well as their mucoadhesive and permeability-enhancing properties (Mao et al., 2010). When encapsulating DNA into nanoparticle compounds, chitosan formulation enhances the integrity of DNA vaccine on the mucosal surface and the uptake of vaccines by mucosal APCs (Smith et al., 2014), thus improves immune induction against mucosal pathogens at the mucus including mucosal T and SIgA responses (Sharma et al., 2015). However, chitosan could hardly precisely direct vaccines to the wanted tissues or cells, leading to insufficient antigen encounter and efficient substance dissipation.

Major challenge in the development of mucosal vaccines is poor transfection efficiency to mucosal epithelial cells and poor immunogenicity of vaccine subunits due to the lack of “danger signals” that can activate local APCs (Kim et al., 2007). Receptor-mediated endocytosis offers advantages with APCs targeting and enhancement of DNA transfection efficiency. Mannose receptors are abundantly expressed on membrane of macrophages and DCs which facilitate recognition and endocytosis of mannose- or fucose-enriched pathogens (Diebold et al., 1999; Yeeprae et al., 2006; Park et al., 2008). The addition of mannose residues on immunogen or vaccine carriers would facilitate the uptake of antigens or particles preferentially by APCs and especially the macrophages (Stambas et al., 2002; Li et al., 2016). Meanwhile, mannose is a good pathogen-associated molecular pattern (PAMP) to stimulate TLR innate response. The modification of mannose to chitosan (Man chitosan, MCS) significantly enhanced the transfection efficiency of the DNA/chitosan complex and reduced its cytotoxicity in macrophages (Peng et al., 2015). MCS-mediated cytokine gene delivery systems led to higher production of the gene in DCs and more efficient induction of IFN-γ from DCs (Hashimoto et al., 2006; Kim et al., 2006). Recently, MCS nanoparticles based Foot and Mouth disease virus (FMDV) DNA vaccine construct was found optimum in inducing the immune response in guinea pigs as measured by FMDV specific neutralizing antibodies and Th1/Th2 responses (Nanda et al., 2014).

In the present study, on base of a previous multi-epitope TB DNA vaccine (pPES) developed by us, we employ mannose-modified chitosan (MCS) nanoparticles to formulate DNA for targeting alveolar macrophages expressing a mannose receptor. We demonstrate here that intranasal immunization of MCS-DNA induces SIgA production in the BAL, and activation of both cytokine-producing CD4+ and CD8+ T cell responses in the lung mucus, which is superior to that by subcutaneous BCG vaccination.

## Materials and methods

### Animal, bacterium, DNA and protein

Female C57BL/6 mice, 6 weeks of age, were purchased from Shanghai SLAC Laboratory Animal Co. Ltd and housed in pathogen-free facility. Animals were cared for in accordance with the Guide for the Care and Use of Medical Laboratory Animals (Ministry of Health, P.R.China, 1998) and animal experiment procedures were approved by the Animal Ethical Committee of Soochow University (SYXK2015-0018). *Mycobacterium bovis* BCG (Denmark strain 1331), provided by the Center for Disease Control of Suzhou, was cultured in Middlebrook 7H9 broth (BD) supplemented with Middlebrook 10% OADC enrichment (Invitrogen), 0.5% glycerol, and 0.05% Tween 80. *M. tuberculosis* H37Rv strain was provided by Fifth People's Hospital of Suzhou and ELISPOT assay using inactivated H37Rv was conducted in ABSL II facility. Recombinant DNA construct, pPES, with 5 T-cell-epitopes from *H37Rv* (MTB10.4_3–11_, ESAT-6_1–20_, Ag85B_241–255_, PPE25_241–255_, and PE19_4–18_) grafted into HSP65 scaffold was prepared by us as previously reported (Wu et al., 2016). Peptides with sequences (ESAT-6_1–20_, MTEQQWNFAGIEAAASAIQG; Ag85B_241–255_, QDAYNAAGGHNAVFN; MTB10.4_3–11_, QIMYNYPAM; PPE25_241–255_, AQFFASIAQQLTFGP; PE19_4–18_, VTTQPEALAAAAANL) were synthesized by GL Biochem Corp (Shanghai, China) with purity over 95%. HSP65 protein was prokaryotically expressed and purified as previously described (Wu et al., 2016). Mannosylated chitosan (MCS) was synthesized from chitosan (CS, MW 390,000; Sigma) by Tianjin Chemsyntech Chemical Technology Co. Ltd. according to the reported procedures (Nanda et al., 2014).

### Preparation and characterization of CS/MCS-DNA formulation

Plasmid DNA (pPES), was amplified in *E. coli* DH5α and isolated with a Qiagen Endotoxin-free Plasmid Giga Kit (Qiagen Inc.) according to the instruction manual provided. Chitosan- or man chitosan-DNA complex was prepared by coacervation as previously described (Roy et al., 1999). Equal volumes of CS or MCS solution (0.02%, 5 mM NaAC-HAc, pH 5.5) and DNA (800 μg/ml in 5 mM Na_2_SO_4_) were vigorously mixed under 55°C for 30 s. The morphology of nanoparticles was examined by scanning electron microscopy (SEM). Size distribution of nanoparticles was detected by light scattering spectrometer (ALV/SP125, ALVGmBH, Langen, Germany) analysis.

### Immunization with MCS-DNA or BCG

Groups of mice (*n* = 6–10) were immunized under anesthesia with CS-vector, MCS-vector, pPES, CS-pPES, and MCS-pPES for 4 times biweekly, each time with 50 μg DNA per mouse. For intranasal (i.n.) delivery of vaccine, freshly made CS- or MCS-DNA complex was delivered into mouse airways in two aliquots with a pipette tip. *M. bovis* BCG at the dose of 10^6^ CFU/mouse was diluted with PBS in a total volume of 100 μl and subcutaneously (s.c.) injected into mice around each hind leg. Bronchoalveolar lavage (BAL) fluid and sera were collected every 2 weeks. BAL fluid was obtained by injecting and recovering two 0.5 mL aliquots of ice cold PBS via a tracheal cannula according to a published protocol (Jhingran et al., 2016).

### Isolation of pulmonary lymphocytes

The lungs were cut into 0.5 cm pieces and subjected to collagenase IV (1 mg/mL, Sigma) and DNase I (5 U/mL, Sigma) digestion for 1.5 h at 37°C with stirring and then mashed through a 70 μm Falcon strainer. Individual cell suspensions from 5 to 6 mice were pooled and cell precipitation was resuspended in 40% percoll (GE Healthcare). Leukocytes were isolated by 40–70% percoll followed by centrifugation for 30 min at 800 g and resuspended in RPMI 1640-10% FCS.

### ELISA assay of HSP65-specific Ab

Plates were pre-coated with 10 μg/ml purified HSP65 protein overnight at 4°C. After blocking with 5% non-fat milk in PBS, 10-fold serially diluted serum or lung lavage were added in duplicate and incubated for 1 h. PBS was used as background control and samples from naïve mice were used as negative control. After incubation with HRP-conjugated goat anti-mouse IgG, and IgA (Southern Biotech) for another 1 h, TMB substrate (eBioscience, USA) was used to develop color and absorbance measured at 450 nm.

### ELISPOT assay

The 96-well filtration plate (Millipore) was pre-coated with 10 μg/ml anti-IFN-γ capture antibody (BD Pharmingen) overnight at 4°C. After washing, the plates were blocked for 2 h with complete RPMI-1640. Splenocytes or pulmonary lymphocytes (5 × 10^5^/well) from individual mice were stimulated for 36 h at 37°C with RPMI 1640 alone, 10 μM single peptide or mixed *M. tb* peptides (ESAT-6_1–20_, MTB10.4_3–11_, Ag85B_241–255_, PPE25_241–255_, and PE19_4–18_), 10 μg/ml HSP65 protein, 5 μg/ml inactivated H37Rv or concanavalin A (5 μg/ml; Sigma-Aldrich) plus anti-mouse CD28 mAb (1 μg/ml; BD Pharmingen). After removing the supernatant, ice-cold deionized water was added and incubated on ice for 10 min to lyse the remaining cells. After washing, biotinylated anti-IFN-γ (BD Pharmingen) and HRP-streptavidin (BD Pharmingen) were added and incubated for 1 h. The enzyme activity was revealed by using AEC substrate kit (BD). Spots were counted by immunospot analyzer KS Elispot software (Carl Zeiss Vision, GmbH).

### Intracellular cytokine staining

To detect intracellular cytokine production, 5 × 10^6^ splenocytes or pulmonary lymphocytes were incubated at 37°C with 10 μM mixed peptides plus 1 μg/ml anti-CD28 mAb for 10 h, and treated with 10 μg/ml Brefeldin A (BD Pharmingen) for an additional 5 h. 1 × 10^6^ cells were first stained with surface markers of PE-Cy7-CD4 or PerCP-CD8(BD Pharmingen) for 30 min, then fixed and permeabilized by Cyto Fix/Perm (BD Pharmingen) for 30 min. Intracellular staining was performed using FITC-IFN-γ, PE-IL-2, APC-TNF-α Abs (BD Biosciences). Flow cytometric data were acquired using a FACSCanto II (BD Biosciences) and analyzed using FlowJo software (TreeStar, San Carlos, CA).

### Pulmonary challenge with mycobacterium BCG

Four weeks after the last immunization, 1 × 10^7^ CFU of *M. bovis* BCG (100 μl in PBS) were delivered into mouse airways. Four and eight weeks post-challenge, bacterial burden was determined by plating serial 10-fold dilutions of lung and spleen homogenates onto Middlebrook 7H11 agar plates containing oleic acid-albumin-dextrose-catalase enrichment (Difco, Detroit, MI). Plates were incubated for 4 weeks at 37°C and colonies were counted.

### Histopathology

Lung and spleen tissues were harvested for histological evaluation 4 and 8 weeks after BCG challenge, fixed in 10% buffered formalin and embedded in paraffin. Sections of 5 μm were stained with hematoxylin and eosin and analyzed by a pathologist who was blinded to the treatment they had received.

### Confocal laser scanning microscopy

CS or MCS was labeled with FITC as described previously (Huang et al., 2002). Mice were i.n. immunized with FITC-CS or FITC-MCS-formulated DNA, and the fresh lung specimens were dissected 24 h later and embedded in Tissue-Tek OCT compound (Bayer Healthcare). Five μm cryostat sections were obtained which were fixed in 4% paraformaldehyde for 30 min, blocked with 1% BSA for 1 h. After incubation with rabbit anti-mouse MOMA (1:100 in 1% BSA, Abcam) overnight at 4°C, TRITC-conjugated goat anti-rabbit IgG antibody (1:100 in PBS, Southern Biotech) was added for 1 h at room temperature. DAPI was used to label nuclei. Confocal fluorescent images were captured using a Nikon A1 confocal microscope.

### Statistical analysis

One-way ANOVA analysis was employed to compare the levels of antigen-specific cytokines, the numbers of polyfunctional T lymphocytes, antibody titers, and organ burdens between the different groups using GraphPad Prism software version 5.01. A *p* < 0.05 was considered significant.

## Results

### Construction and biochemical property of MCS-pPES nanoparticle

**Figure 1:**
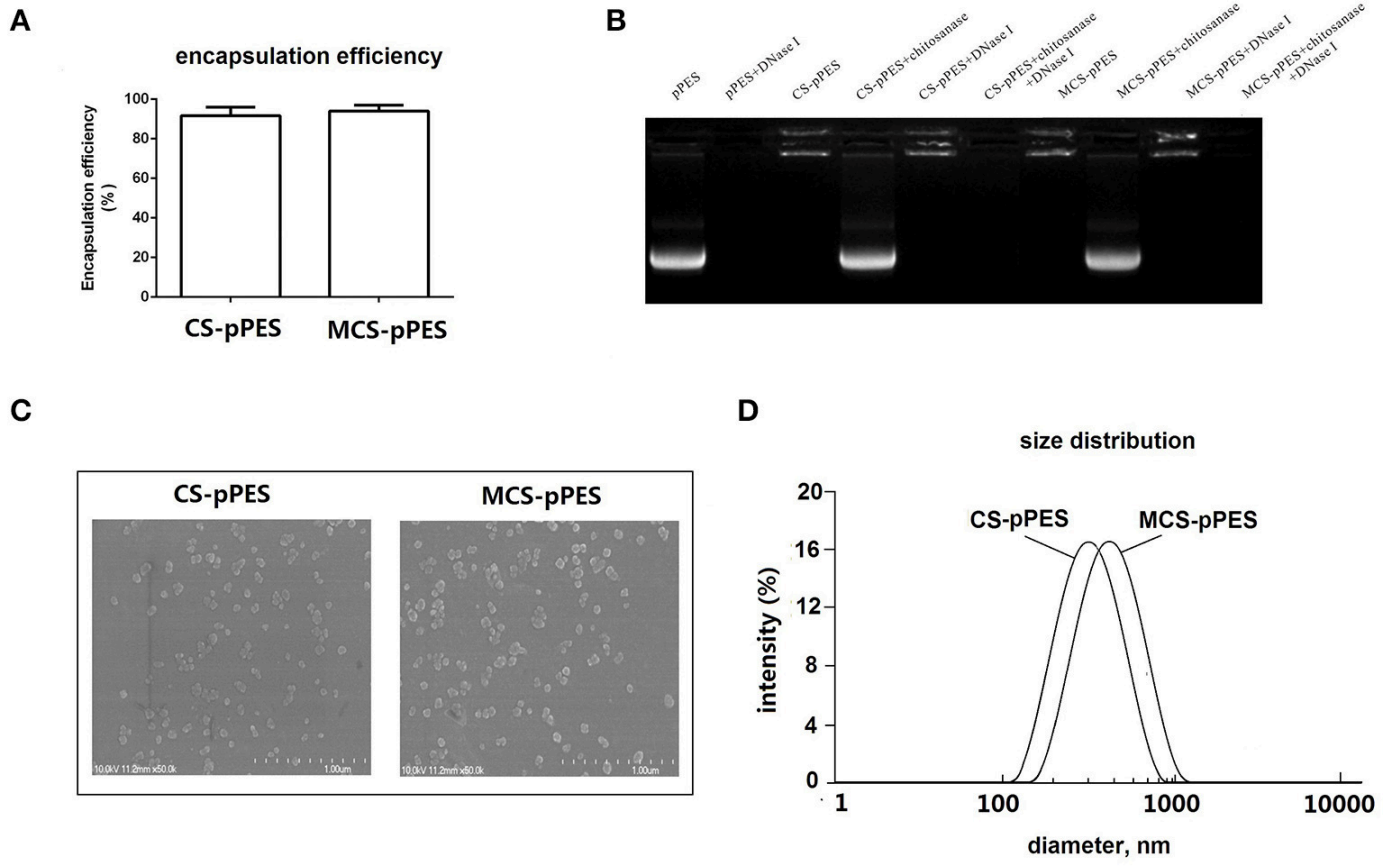
Preparation and characterization of MCS-pPES nanoparticle. **(A)** DNA encapsulation efficiency of MCS-pPES formulation. **(B)** Electrophoretic mobility analysis of MCS-pPES or CS-pPES nanoparticles following DNase I digestion. **(C)** The scanning electron micrograph of freshly prepared CS- or MCS-pPES complexes. Scale bar: 1,000 nm. **(D)** Particle size distribution detected by DLS analysis.

MCS-pPES and CS-pPES particles were prepared by co-acervation method, and similar high DNA encapsulation efficiencies (>90%) were demonstrated by spectrophotometry assay (Figure 1A). Then MCS-DNA nanoparticles were characterized for gel retardation assay, physicochemical characteristics and dynamic DLS assay. Both MCS and CS could efficiently protected DNA from DNase I digestion (Figure 1B). Transmission electron microscopy revealed that both MCS- and CS-DNA formulation appeared spherical and compact structures (Figure 1C). By using dynamic light scattering, the average hydrodynamic radius of CS-DNA and MCS-DNA particles were about 300 and 400 nm, respectively (Figure 1D). These data suggested that mannose modification of chitosan did not influence the encapsulation efficiency and the physicochemical characteristics of CS formulated nanoparticles.

### Intranasal immunization with MCS-pPES enhanced bronchoalveolar lavage SIgA induction

**Figure 2:**
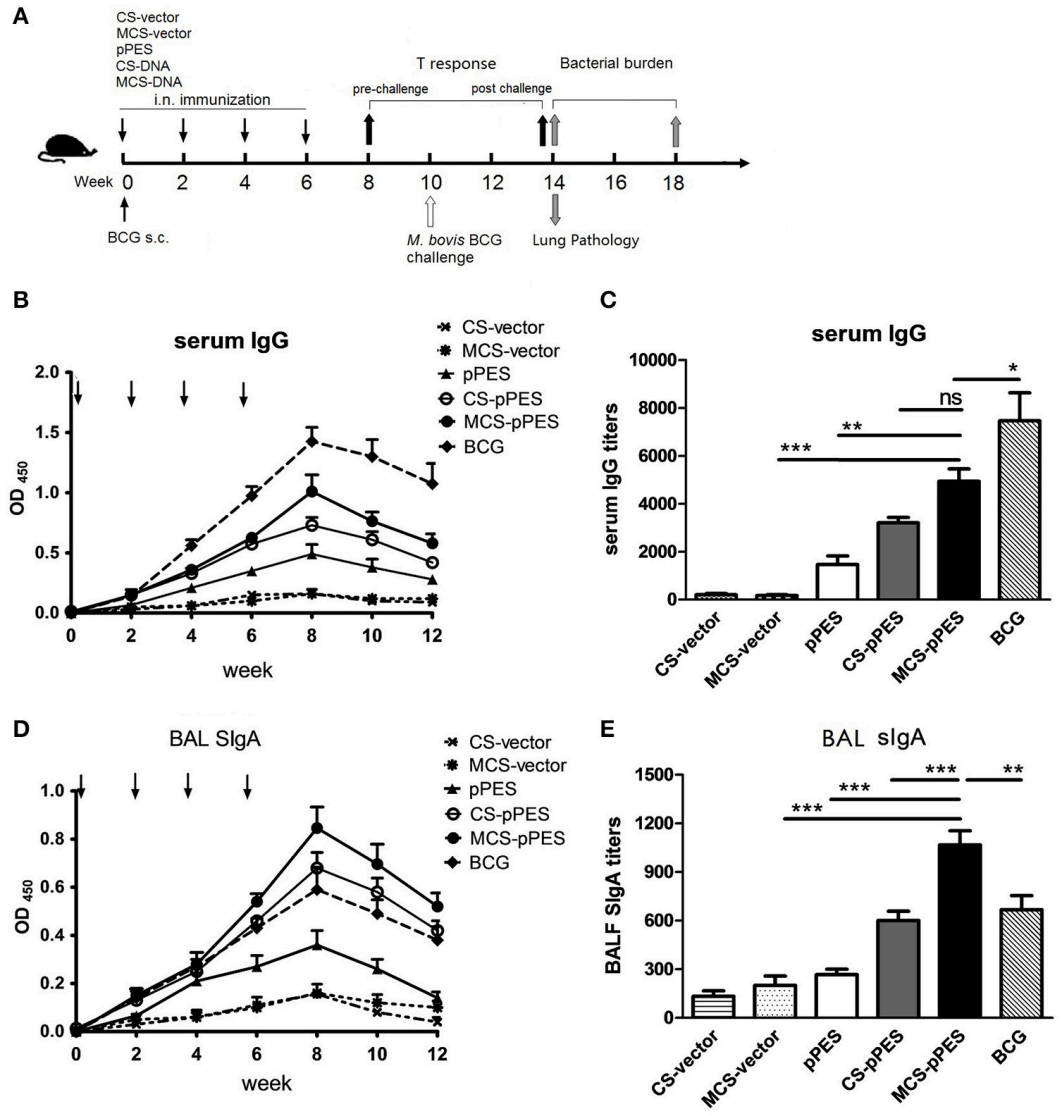
MCS-pPES intranasal immunization significantly enhanced HSP65-specific bronchoalveolar lavage fluid SIgA induction. **(A)** Schematic diagram of the time course of animal immunization and challenge regimen. Mice were intranasally immunized with 4 doses of MCS- or CS-DNA. One dose of 10^6^ CFU BCG was subcutaneously injected into hind legs of mice. Levels and titers of HSP65-specific serum IgG **(B,C)** and bronchoalveolar lavage fluid SIgA **(D,E)** were detected by ELISA assays. Data are representative of three independent experiments and presented as the mean ± SEM (*n* = 6). ^*^p < 0.05; ^**^p < 0.01; ^***^p < 0.001; ns, not significant.

To evaluate the capacity of MCS formulated DNA to induce HSP65-specific antibodies, C57BL/6 mice were intranasally immunized with 4 doses of MCS-pPES vaccine biweekly (Figure 2A), serum IgG and BAL SIgA were examined by ELISA. HSP65-specific serum IgG and BAL SIgA levels induced by all vaccines increased gradually after immunization, with the highest response observed 2 weeks after the last immunization (Figures 2B,D). Compared to CS-DNA and naked DNA, intranasal MCS-DNA induced a significantly elevated level of serum IgG (*p* < 0.01, Figure 2C), although still much lower that induced by BCG s.c. injection. However, the HSP65-specific BAL SIgA level induced by intranasal MCS-DNA was significantly higher than that triggered by CS-DNA, and BCG (titer, 1,100 vs. 860 and 700, *p* < 0.01, Figure 2E). Thus, compared to BCG immunization, intranasal mannosylated chitosan-formulated DNA elicited a significantly increase in SIgA response in the respiratory tract.

### Intranasal vaccination with MCS-DNA elicited modest Th1 immune responses in the spleen

**Figure 3:**
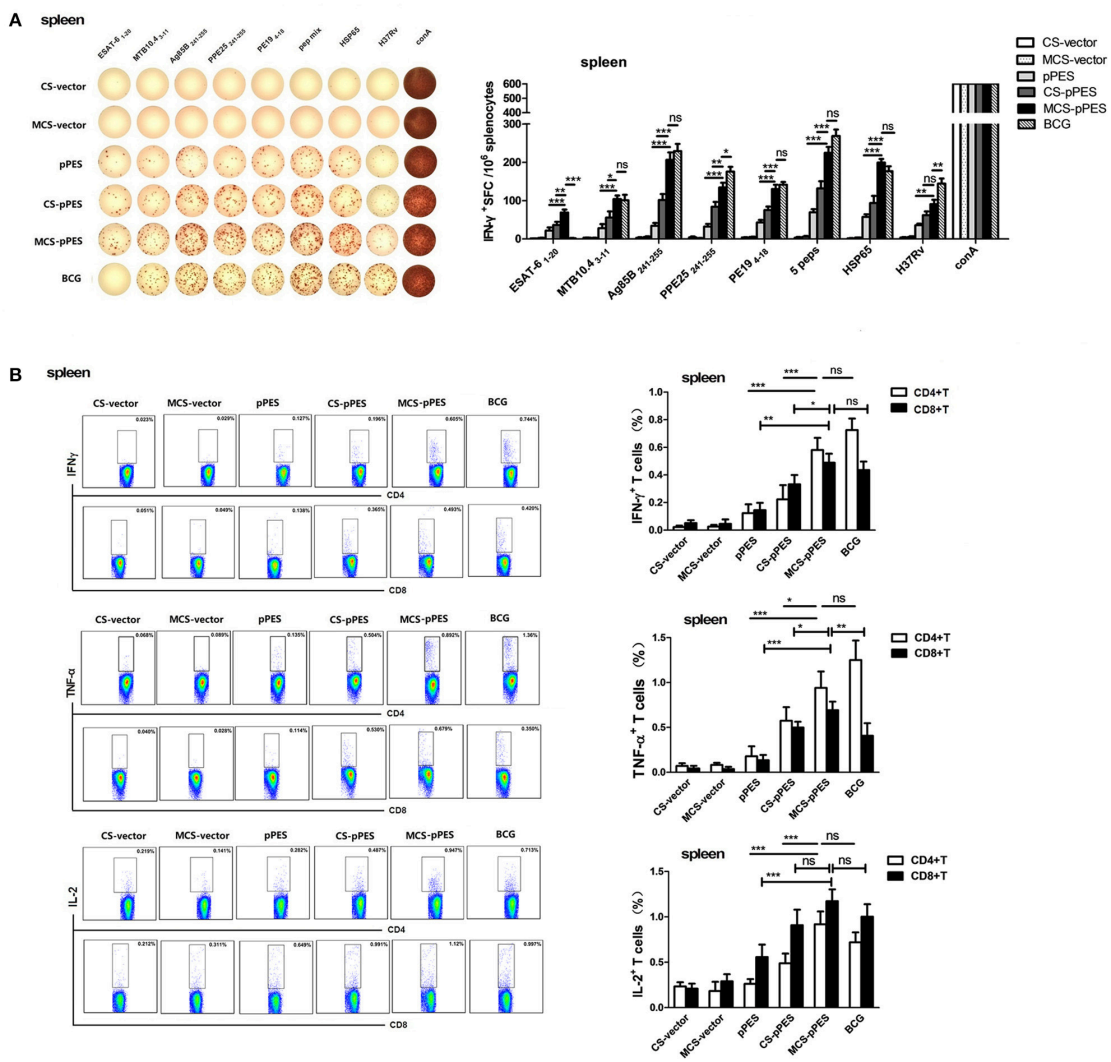
Intranasal immunization with MCS-pPES enhanced specific IFNγ-secreting CD4+ and CD8+ T response in the spleen. **(A)** ELISPOT analysis of the splenic TB-specific IFNγ-producing T response in pPES-, CS-DNA, MCS-DNA-, and BCG-immunized mice. Splenocytes were stimulated with mixed peptides, HSP65 protein or inactivated H37Rv plus anti-CD28 for 36 h before IFN-γ ELISPOT assay was performed. Statistical results were from three independent experiments and presented as the mean ± SEM (*n* = 6). **(B)**The frequency of CD4 and CD8 T cells in the lung producing IFN-γ, IL-2, or TNF-α in response to mixed peptides was measured. Cells were incubated with mixed peptides plus anti-CD28 for 10 h the percentages of IFN-γ, IL-2, and TNF-α-producing CD4+T and CD8+T cells were determined by FACS. Numbers in each quadrant represent percentages of positive cells in CD4+ or CD8+ T population. Results are represented as mean ± SEM (*n* = 6) of three separate experiments. ^*^p < 0.05; ^**^p < 0.01; ^***^p < 0.001; ns, not significant.

First, we investigated how the intranasal MCS-DNA vaccine would affect the magnitude and quality of antigen-specific T cell response in the spleen. First, ELISPOT assays were performed to investigate specific IFN-γ production and peptide responsibility of T cell responses. All vaccines activated T response specific to the peptides mixture and HSP65 protein; however, only BCG vaccine activated T cells responding to the inactivated H37Rv. Among the five peptides, T cell response to ESAT6_1–20_ and MTB10.4_3–11_ were much lower than that to the other 3 epitope peptides following all vaccination. MCS-pPES induced IFN-γ+ T response to ESAT-6_1–20_, which could not be induced by BCG (Figure 3A). FACS assay showed that intranasal MCS-DNA induced a significantly enhanced five peptide mixture-specific IFN-γ+, TNF-α+, and IL-2+ T response (CD4+ and CD8+) than CS-DNA and DNA vaccine (Figure 3B), the level comparable to or less than that induced by i.d. BCG vaccine (*p* > 0.05). Thus, intranasal MCS-pPES elicited a modest M.tb specific CD4+ and CD8+ T response in the spleen.

### Intranasal vaccination with MCS-DNA elicited a marked elevated multi-functional T immune response in the lung

**Figure 4:**
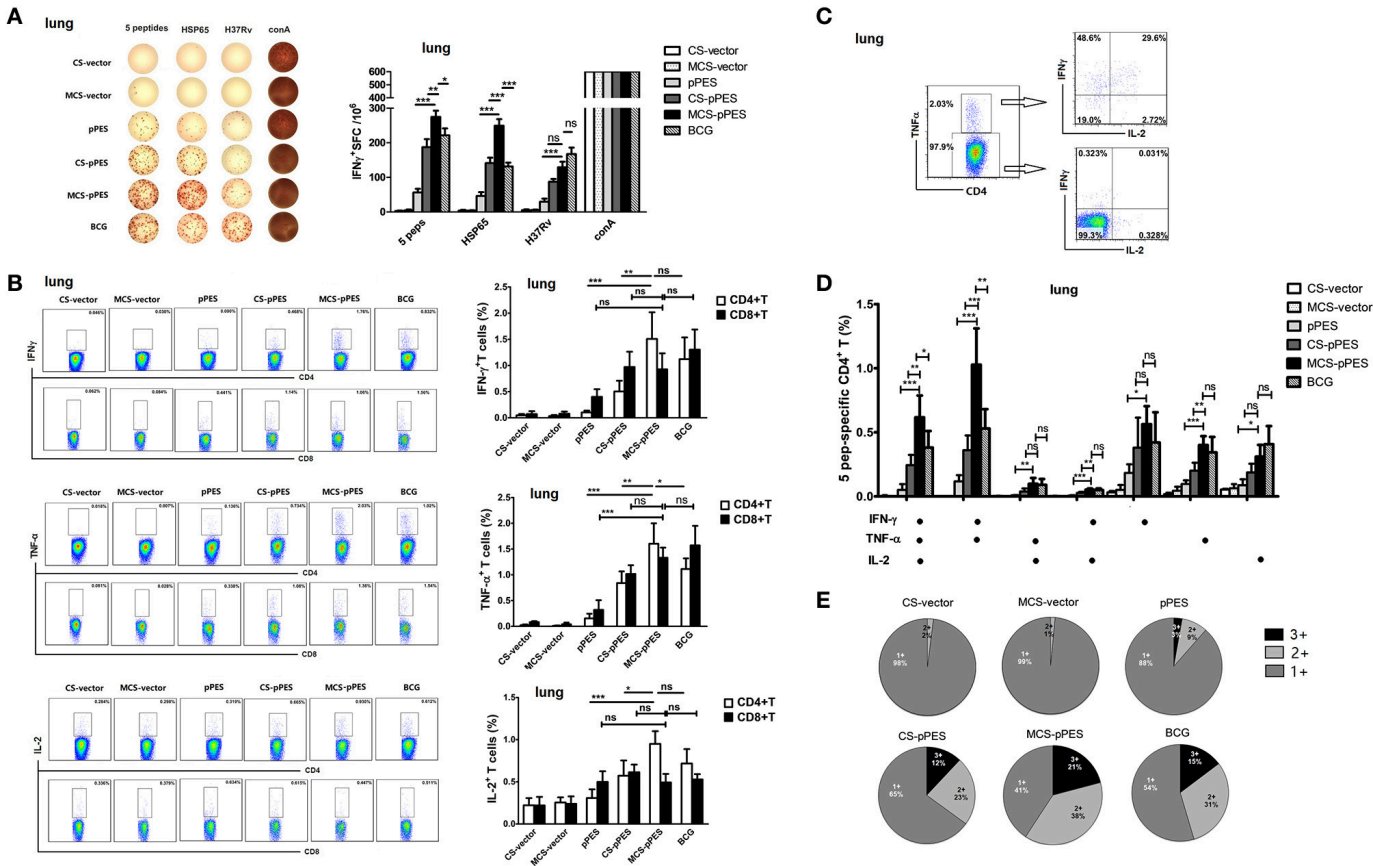
MCS-pPES intranasal immunization enhanced a poly-functional Th1 response in the lung. **(A)** 2 weeks after final immunization, pulmonic lymphocytes were stimulated with mixed peptides plus anti-CD28 for 36 h, and specific IFNγ-producing T cell spots were determined by ELISPOT assay. A representative result was shown. Statistical results were from three independent experiments and presented as the mean ± SEM (*n* = 6). **(B)** The frequency of CD4 and CD8 T cells in the lung producing IFN-γ, IL-2, or TNF-α in response to mixed peptides was measured by FACS. Numbers in each quadrant indicate percentages of positive cells in CD4+ or CD8+ T population. Results are represented as mean ± SEM (*n* = 6) of three separate experiments. **(C)** CD4+TNF-α+ and CD4+TNF-α- cells within CD4+ T cells were further gated, and the expression of IFNγ and IL-2 was shown. **(D)** The total peptide-specific Th1 response within CD4+ T cells was defined as the number of cells expressing any combination of IFN-γ, TNF-α or IL-2. The average percentages for each subset are shown. Results are represented as mean ± SEM (n = 6) of three separate experiments. **(E)** The quality of the T cell responses in **(C,D)**. Pie charts showed the fraction of total cytokine-response in the lung comprising any combination of IFN-γ, IL-2, or TNF-α. The percentages of cells producing three cytokines (triple positive), two cytokines (double positive) or one cytokine (single positive) within the total CD4+T cell response were shown from three independent experiments. ^*^p < 0.05; ^**^p < 0.01; ^***^p < 0.001; ns, not significant.

The primary objective of this study was to determine whether a MCS-formulated DNA vaccine delivered by intranasal route would elicit robust and protective TB-specific T cell cytokine responses in the lung, the initial site of TB infection. We assessed by ELISPOT assay the TB-specific IFN-γ-secreting cells in the lungs of mice. Although intranasal MCS-DNA vaccine induced a relative weak IFN-γ-T response to inactivated H37Rv, it produced significantly stronger IFN-γ-T responses (275, 249 SFC/10^6^) to mixed peptides or HSP65 protein; which were significantly higher than those induced by CS-DNA (187, 141 SFC/10^6^, *p* < 0.01) and by BCG (222, 132 SFC/10^6^, *p* < 0.05, Figure 4A).

The quality (cytokine expression profile) of the vaccine-elicited T cell response has implications for disease outcome in TB. Thus, we assessed the cytokine profile of CD4+ and CD8+ T cell response in the lungs prior to challenge by intracellular cytokine staining. Concerning single cytokine T response, intranasal MCS-DNA induced potent peptide-mixture-specific CD4+ and CD8+ effector T cells in the lungs of mice that largely produced TNF-α and IFN-γ, as well as IL-2. And single TNF-α or IFN-γ responses by CD4+ T cells induced by MCS-DNA were significantly higher than those induced by CS-DNA (*p* < 0.01, Figure 4B) and were comparable to that induced by BCG. We further assessed the quality of CD4+ T response and found that MCS-DNA-induced CD4+T response in the lungs was dominated by TNF-α+IFN-γ+ double positive response (38%) and ~21% of the CD4+ T cells produced IFN-γ, TNF-α, and IL-2 simultaneously (Figures 4C–E). Compared with response induced by BCG vaccine, specific CD4+ T cells by MCS-DNA were comprised of more triple-positive cells (21 vs. 15%) and a higher proportion of IFN-γ and TNF-α double-positive (38 vs. 31%) and IFN-γ single-positive cells (17 vs. 12%) (Figure 4E). These data indicated that intranasal man-chitosan encapsulated DNA was more efficient to elicit potent multifunctional T response in the lung than BCG vaccination.

### Protection efficacy of intranasal MCS-pPES in lungs and spleens of mice after BCG challenge

**Figure 5:**
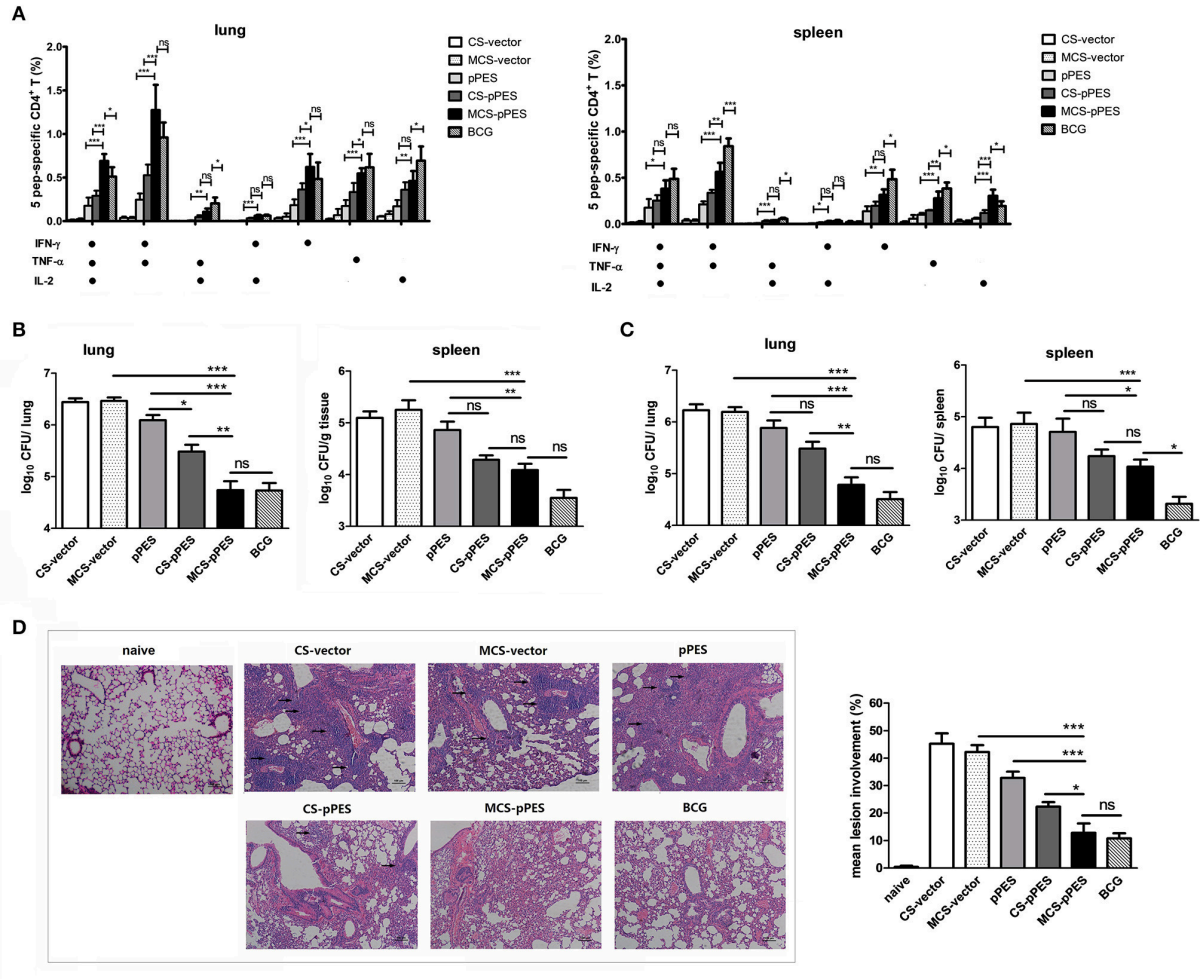
Post-challenge T response and protection against *Mycobacterium bovis* BCG challenge by MCS-pPES immunization. Vaccinated mice were challenged i.n. with 1 × 10^7^ CFU of *M. bovis* BCG**. (A)** 4 weeks post-challenge, the peptide-specific poly-functional CD4+Th1 responses in the lungs or spleens were defined as the number of cells expressing any combination of IFN-γ, TNF-α or IL-2. The average percentages for each subset are shown. Results are represented as mean ± SEM (*n* = 3) of three separate experiments. **(B,C)** Bacterial load within the lungs and spleens of mice 4 weeks **(B)** and 8 weeks **(C)** post-challenge with BCG. Results are presented as log_10_CFU within each organ. Data are expressed as mean ± SEM from pooled three separate experiments (*n* = 6). ^*^p < 0.05; ^**^p < 0.01; ^***^p < 0.001; ns, not significant. **(D)** Representative lung pathology of mice by H&E staining 4 weeks after challenge, showing the immune infiltration and granuloma-like pathology in the lung (denoted by black arrows). One representative image of each group of three separate experiments was shown (magnification: × 100). Inflammation lesion involvement within the lungs of mice was summarized. Data are represented as mean ± SEM (*n* = 5). Three separate experiments were repeated and one result was shown.

Four weeks after the final immunization, vaccine efficacy was tested in mice challenged intranasally with 1 × 10^7^ CFU of BCG. First, we assessed the post-challenge multifunctional T response in the lungs and spleens of mice. IFNγ/TNFα double-positive cells responding to peptides still comprised the majority of the Th1 cytokine-producing CD4+T cells. Similar to pre-challenge T cell response, CD4+ T cells in lungs of MCS-DNA-immunized mice produced significant more amounts of triple cytokines than CD4+T cells from CS-DNA (*p* < 0.001) and BCG-vaccinated (*p* < 0.05, Figure 5A) mice did. However, BCG challenge recalled a more potent post-challenge IFNγ/TNFα/IL-2 triple-positive and IFNγ/TNFα double-positive CD4+ Th1 response in spleens of BCG-treated mice as compared to those of MCS-DNA-vaccinated mice (Figure 5A).

The bacterial burdens were evaluated 4 and 8 weeks after challenge. At 4 weeks post-challenge, compared to naked DNA immunization, CS-DNA significantly reduced lung bacterial CFUs, and intranasal MCS-DNA vaccination further led to a 0.7 Log reduction in lung bacterial CFUs (*p* < 0.01), effect comparable to BCG vaccination (Figure 5B). However, although MCS-DNA immunization led to a 0.8 Log reduction of infection in the spleen as compared to DNA vaccine (*p* < 0.01), still single s.c. BCG vaccination conferred the greatest extent of protection in the spleen, with infection reduced by 1.3 Log (*p* < 0.01, Figure 5B). At 8 weeks after challenge, MCS-DNA significantly reduced CFU in the lungs by 1.4-log10 compared to MCS-vector controls (*p* < 0.001, Figure 5C), effect a little lower than but comparable to that of BCG vaccination (1.7-log10 reduction in CFU, *p* > 0.05 as compared to MCS-DNA). The week 8 bacterial burden in spleens showed similar pattern to CFU in lungs but the best protection was achieved by BCG vaccination (CFU reduction by 1.55-log10, *p* < 0.05 as compared to MCS-DNA vaccine).

Consistent with varying bacterial loads in the lung, HE-stained lung sections from CS- and MCS-vector control mice showed severe interstitial pneumonia and intense inflammation throughout the lung with early granuloma-like pathology (lesion burdens ranging from 42 to 45%) after *M. bovis* BCG challenge (Figure 5D). Less but still intensive damage in alveolar tissues with aggregated lymphocytes was observed in the lung from DNA-vaccinated group. Compared to DNA vaccine, chitosan formulation significantly improved protection showing limited interstitial pneumonia. The MCS-DNA and BCG groups showed significantly less lesion involvement (12.8 and 10.8%, *p* < 0.001 as compared to DNA and MCS-vector group), almost intact alveolar morphology and more evidence of protection within the lungs 4 weeks post-challenge (Figure 5D). These results suggest that i.n. MCS-DNA immunization confers improved protection in the lung over i.n. CS-DNA and DNA vaccine that is comparable to that achieved by s.c. BCG vaccination.

### Enhanced alveolar macrophage targeting by MCS formulation

**Figure 6:**
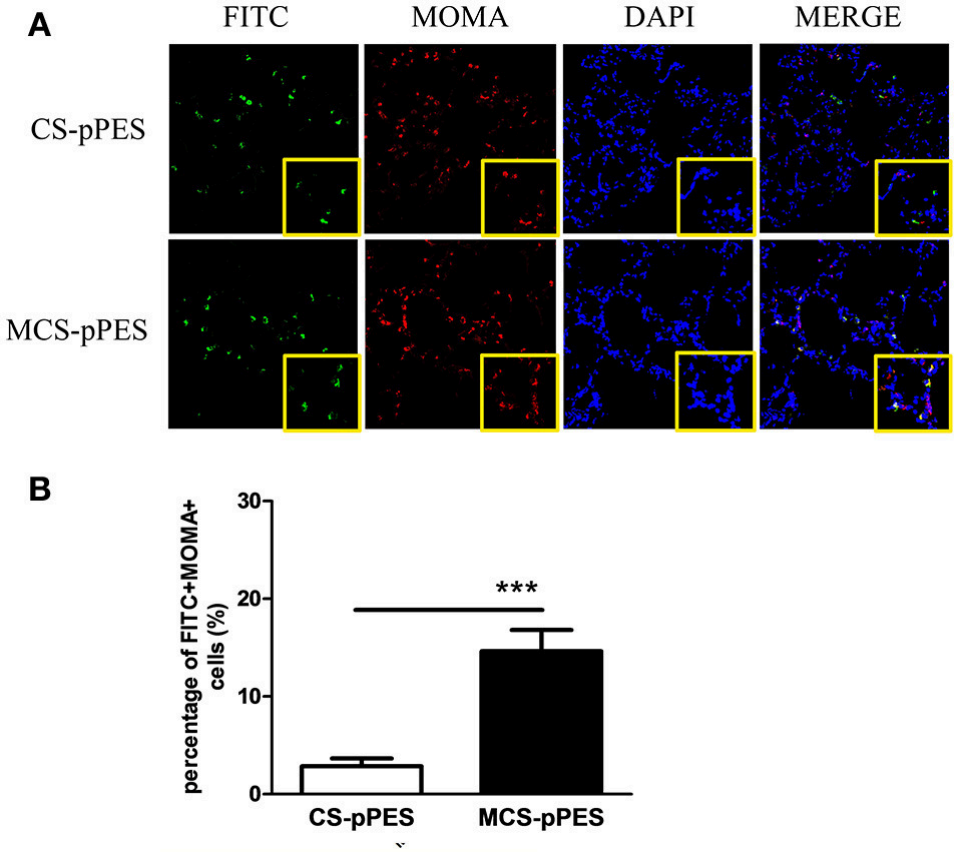
Increased alveolar macrophage targeting property of MCS-pPES Vaccine. **(A)** 24 h after intranasal FITC-CS- or FITC-MCS- DNA immunization, lungs of mice were OCT-embedded before freezing and cyrosections (5 μM) were subjected to fluorescent staining. Composite confocal represented images of lung cyrosections 24 h after immunization showing FITC (green), MOMA (red), and nuclear stain with DAPI (blue). Low magnification (magnification, × 200) and higher magnification of the boxed areas (magnification, × 400) are shown. **(B)** The number of DNA-accessing alveolar macrophages in the lung sections of vaccine-immunized mice were numerated as a percentage of FITC+MOMA+cells in MOMA+ cells. Data are expressed as mean ± SEM from three repeated experiments (*n* = 3). ^***^p < 0.001.

To explore the mechanism of MCS-DNA formulation in improving immune induction in the lung, the accessibility of man-chitosan-DNA complex to alveolar macrophages was visualized by a confocal laser scanning microscope on lung cyrosections from 24 h FITC-CS-DNA and FITC-MCS-DNA-immunized mice. At 24 h, although comparable levels of DNA got access to the alveoli, an increased co-localization of DNA (FITC+, green) with AMs (MOMA+, red) was observed in the lung cryosections of MCS-DNA-immunized mice than in those of CS-DNA-treated mice (Figure 6A). Quantification of frequency of alveolar macrophages both positive for DNA staining from independent fields indicated that percentage of FITC+MOMA+ cells within all MOMA+ alveolar macrophages in lung sections of MCS-DNA-treated mice (14.6%, *p* < 0.001) was significantly higher than that (2.7%) seen in lung sections of CS-DNA-immunized mice (Figure 6B). Taken together, these data supported that mannose modification of chitosan enhanced vaccine antigen direction to the alveolar macrophages which would favor the local antigen presentation and immune induction in the lung.

## Discussion

Development of an ideal vaccine against pulmonary tuberculosis (TB) may require immunization strategies that generate a high frequency of antigen-specific CD4 and CD8 T cell response in the periphery and in the lung (Darrah et al., 2014). Traditional subcutaneous (s.c.) BCG immunization predominantly elicits low PPD-specific T responses, and undetectable T response to Ag85A/B in the BAL (lung) (Darrah et al., 2014), which may explain variability in the protection afforded by BCG in humans. Mucosal vaccination can induce antigen-specific humoral and cell-mediated immune responses in both the systemic and mucosal compartments which also include long-lasting B- and T-cell memory response (Lycke, 2012). Intranasal vaccination is reported effective for the immune induction in respiratory, gastric and genital tracts, especially. Thus, we hypothesize that the intranasal route and alveolar macrophage-targeting mucosal delivery strategy would most directly and effectively induce T cell immunity in the lung and thereby improve protection against pulmonary TB. Out data showed that intranasal delivery of a man-chitosan-formulated DNA vaccine expressing 5 epitopes from ESAT-6, Ag85B, TB10.4, PPE19, and PPE25 induced a potent and enhanced BAL SIgA response, and peptide-specific multi-cytokine-producing effector CD4 and CD8 T responses in the lung mucosa of mice as compared to naked, chitosan-formulated DNA and BCG vaccine. These responses indeed conferred ameliorated lung pathology and decreased bacterial burdens in the lung following challenge compared with effect of DNA and chitosan-DNA vaccine, although the peripheral (spleen) protection was not significantly improved.

Modulation of immunity, especially manipulating antigens to be more efficiently presented by antigen-presenting cells (APCs) that can activate the effector T and B cells of the immune defense is a major goal in treatment and prevention of bacterial or viral infections (Lai et al., 2015). The respiratory tract is the portal of entry for *M.tb* (Rodriguez et al.). Alveolar macrophages (AMs) not only serve as the initial major host cell niche for the growth and survival of *M.tb*, but also constitute the first line of innate defense against *M.tb* and represent local APCs responsible for activation of protective T and Ab responses (Weiss and Schaible, 2015). One possible reason that BCG and other parenteral TB vaccine candidates fail to contain *M.tb* infection is their inability to provoke an effective and sustained innate response as well as antigen presentation in the lung before a much delayed T cell response is induced (Kim and Jang, 2017). In this regard, manipulating the APC function of AMs or targeting antigen more efficiently to AMs represents attractive strategy to enhance the efficacy of TB vaccines. Macrophages and immature DCs express high levels of mannose receptor (MR) which is critical for endocytosis and phagocytosis of mannose-exposed pathogen antigens and participating in antigen processing and presentation, cell migration and intracellular signaling (Loke et al., 2016). And mannose density has profound influence on the cellular uptake and RNAi efficiency of polymeric nanoparticles in macrophages (Chu et al., 2015). Conjugation of mannose residues to vectors or various forms of antigens, would target antigens to endocytic mannose receptors on APCs and promote antigen uptake by MR, thus inducing efficient gene delivery to APCs and enhancing Th1 and CTL response (Jabbal-Gill et al., 2012). Chitosan, a polycationic homopolymer of glucosamine manufactured by the deacetylation of chitin, has being studied as mucosal carrier and an adjuvant in DNA and protein-based vaccines (Sedaghat et al., 2014; Smith et al., 2014). Our previous efforts also demonstrated that respiratory immunization with a chitosan-formulated DNA vaccine enhanced anti-*M.tb* protection via enhancing pulmonary IFN-γ+ T response and SIgA production in BAL fluid (Ai et al., 2013). In this study, to deliver chitosan-DNA complex into alveolar macrophages, we employed mannose-modified chitosan to formulate and target DNA vaccines to the AMs and DCs expressing MR in the lung. Compared to chitosan-DNA, our data demonstrated that man-chitosan formulation of DNA significantly enhanced induction of specific BAL SIgA response (Figures 2C,D) and poly-functional CD4+ T and CD8+ T response at lung mucosa (Figure 4). And, by a confocal laser scanning microscopy on lung cyrosections of mice, we did confirm a significantly increased co-localization of DNA with AMs 24 h following man-chitosan-DNA i.n. vaccination (Figure 6). In addition to our data, previous study showed that mannosylated polymer nanoparticles specifically and effectively delivered siRNA to tumor associated macrophages (TAMs) (Ortega et al., 2015) and made the encapsulated siRNA more capable to manipulate NF-κB signaling in the tumor (Ortega et al., 2016); and man-chitosan nanoparticles effectively enhanced the transfection efficiency of DNA and lowered the toxicity of chitosan in peritoneal macrophages (Hashimoto et al., 2006). Taken together, we and others demonstrate that mannosylation of drugs, proteins, genes or their delivery vectors significantly enhance their accessibility to and transfection efficiency in the macrophages, therefore may greatly enhance the therapeutic efficiency or vaccine immunogenicity.

Apart from macrophage-targeting strategies by mannose modification of antigens, various other APCs may represent the ideal targets of mucosal vaccines. Intestinal M cell-specific markers like GP2 and C5a receptor can be utilized for antigen delivery to gut mucosal immune inductive sites (Wang et al., 2014); Targeting intestinal DC through DEC-205 provides attractive strategy for improving the intestine APC adjacency (Dhodapkar and Dhodapkar, 2014). Glucan particles (GPs)-vectored vaccine antigen favores phagocytosis of GP-Ag via dectin−1 and stimulates cytokine production by myeloid DCs (Hua et al., 2015).

Recent evidence suggest that TB-specific multifunctional, high-level cytokine-producing T cell responses within the airway lumen and lung interstitium (poised at the appropriate anatomical location to protect against respiratory infection) correlate better with protection against TB challenge in mice. Whereas, those detected in blood or lymphoid organs do not correlate well with protection (Forbes et al., 2008). However, in this study, despite intranasal MCS-DNA vaccine induced a robust and an enhanced multi-functional CD4+ T cell response to peptide mixture in the lung prior to or after challenge as compared to BCG vaccine, such response failed to confer overall enhanced protection (compared to BCG) following challenge. MCS-DNA-induced lung response led to a comparable reduction in lung bacterial CFUs as did BCG-vaccination, but failed to reduce the bacterial infection in the spleen as efficiently as BCG immunization did at 4 or 8 weeks after challenge (Figures 5B,C). Generating responses in both the lung and the periphery should be optimal for protection (Darrah et al., 2014). We propose that although i.n. delivery of MCS-DNA is superior to induce a high frequency of lung-resident effector T cells that might protect early after TB infection; its potential to generate a reservoir of peripheral effector or memory cells which can be recruited into lung at later phases of infection is less ideal than that of systemic BCG immunization. And subcutaneous BCG-induced poly-functional splenic Th1 response persisted longer than that induced by intranasal MCS-DNA (Figure 5A), thus could be more effectively recalled by BCG challenge and displayed more potent anti-bacterial protection in the spleens of mice at 8 weeks post-challenge (Figure 5C). Furthermore, high responsibility to peptides but lack of a robust response to H37Rv antigen in MCS-DNA-vaccinated spleens was notable in this study (Figure 3A). This indicates that the five T epitopes incorporated by pPES DNA vaccine are likely not sufficient to mediate overall protection. Therefore, as with other infections (e.g., polio virus) that require whole attenuated organisms for protective cellular immunity, vaccines against TB that incorporate many, rather than few or single antigens might have a greater chance of efficacy. In addition, murine airway luminal *M.tb* antigen-specific CD8+ T effector response induced by intranasal immunization was found long sustained and could confer anti-tuberculosis protection independent of CD4+ T and peripheral T cells (Jeyanathan et al., 2010). In our study, intranasal MCS-DNA elicited a stronger Th1 cytokine-producing CD4+ T response in the lung than BCG; while the IFNγ and TNF-α production by CD8+ T cells from MCS-DNA-immunized mice was less efficient that that from BCG-vaccinated mice (Figure 4B). This lower quality of lung CD8+ T response may also explain the suboptimal protection effect by MCS-DNA vaccine. And the protection by MCS-formulated nasal vaccines would be anticipated to benefit from a heterologous BCG prime-mucosal vaccine boost immunization regimen which helps to generate robust short-term effector responses in the lung and increase the magnitude of BCG-primed T cell responses in the periphery.

At the mucosal surfaces, the predominant immunoglobulin is secretory IgA (SIgA) that specifically neutralizes viruses and prevents bacterial colonization. SIgAs are more and more suggested to have a protective role against viral and bacterial infections like HIV (Sholukh et al., 2015), influenza virus (Morokutti et al., 2014), and, tuberculosis (Alvarez et al., 2013). IgA-/- and pIgR-/- mice were more susceptible to BCG infection compared to WT mice with reduced IFN-γ and TNF-α levels in the lung (Rodriguez et al., 2005). It suggests IgA may play an important role in protection against mycobacterial infections in the respiratory tract by blocking pathogen entrance and/or by modulating the pro-inflammatory responses. Antibodies against surface Ags are now understood to confer protection against tuberculosis by modulating T and macrophage immunity via Fc-receptor mediated phagocytosis (Jacobs et al., 2016). Our data showed that nasal MCS-DNA vaccine induced a significantly elevated BAL SIgA which may contribute to the significant reduction of bacterial CFUs in the lung as compared with CS-DNA. Whether SIgA could opsonize *M.tb* for targeted FcR-mediated phagocytosis, and the importance of respiratory SIgA induction as a primary goal of TB vaccine development need extensive further evaluation.

In the present study, we have provided the evidence that intranasal MCS-DNA immunization was effective to induce a robust and an enhanced BAL SIgA response as well as a poly-functional CD4^+^/CD8^+^T responses in the lung as compared with CS-DNA and systemic BCG vaccination. Although a comparable improved protection was achieved by MCS-DNA intranasal immunization in the lung but not the spleen as compared to BCG s.c vaccination, our data demonstrate that formulation of DNA into mannose-chitosan particles enables preferential vaccine delivery to alveolar macrophages which favors the promoted induction of antigen-specific SIgA and T response in the lung following intranasal immunization. Our data indicate that MCS formulation represents an ideal mucosal system for targeting alveolar macrophages thus favoring stronger antigen-specific T cell response induction directly at the site of TB infection and improving mucosal immune protection against pulmonary TB.

## Author contributions

WX: Conceived and supervised the project, wrote the manuscript. MW and HZ: Performed the experiments. SX: Supervised the project. ML and YY: Interpreted data and participated discussion. All authors approved the final version of the paper.

### Conflict of interest statement

The authors declare that the research was conducted in the absence of any commercial or financial relationships that could be construed as a potential conflict of interest.
